# The oral-gut-circulatory axis: from homeostasis to colon cancer

**DOI:** 10.3389/fcimb.2023.1289452

**Published:** 2023-11-08

**Authors:** Sofia C. Tortora, Maria Gonzalez Agurto, Laura A. Martello

**Affiliations:** ^1^ Department of Medicine and Division of Gastroenterology & Hepatology, SUNY Downstate Health Sciences University, Brooklyn, NY, United States; ^2^ Departamento de Rehabilitación Craneofacial Integral, Universidad de Los Andes, Santiago, Chile

**Keywords:** microbiota, immune response, dysbiosis, oral-gut axis, circulation, colon cancer

## Abstract

The human microbiota is widely recognized as providing crucial health benefits to its host, specifically by modulating immune homeostasis. Microbial imbalance, known as dysbiosis, is linked to several conditions in the body. The oral cavity and gut host the two largest microbial communities playing a major role in microbial-associated diseases. While the oral-gut axis has been previously explored, our review uniquely highlights the significance of incorporating the circulatory system into this axis. The interaction between immune cells, inflammatory factors, circulating bacteria, and microbial metabolites influences the homeostasis of both the oral and gut microbiota in a bidirectional manner. In this comprehensive review, we aim to describe the bacterial components of the oral-gut-circulatory axis in both health and disease, with a specific focus on colon cancer.

## Introduction

Disturbance of the microbiota homeostasis has emerged as a significant factor correlated with a multitude of diseases within the human body. The study of the microbiome and its association with cancer has primarily focused on an organ-specific relationship, however, increasing evidence underscores the pivotal role played by the microbial and inflammatory milieu at distant anatomical sites as an important regulator in both healthy and pathological processes.

The oral cavity and gut harbor the two largest microbial habitats in the body and play a significant role in infectious diseases and in different types of cancer. The mouth is the entry point of the digestive tract and is continuously exposed to various exogenous element, including microorganisms, nutrients, and xenobiotics. Bacterial translocation can occur through different routes, such as swallowing, aspiration and through circulation or transported inside immune cells. Certain oral pathogens, such as *Porphyromonas gingivalis (P. gingivalis)* and *Fusobacterium nucleatum (F. nucleatum)*, have been found to invade and colonize the oral epithelial cells and periodontal tissues where the bacteria can release virulence factors and toxins that disrupt the integrity of the oral mucosa. Additionally, chronic oral infections, such as periodontitis, can weaken the barrier function facilitating the translocation of oral pathogens into the bloodstream, ultimately reaching distant sites such as the liver, spleen, and gastrointestinal tract. In the liver, oral microbes can trigger a low-grade inflammatory reaction and impact various body sites through the release of cytokines. Once in the gut, these oral bacteria can interact with the existing gut microbiota, influencing its diversity and functionality. Changes in the microbial communities may trigger immune responses, inflammation, and increased intestinal permeability, allowing opportunistic bacteria to invade, and toxins and metabolites to leak into the bloodstream. Disturbances in either the oral or gut microbiota can cascade into the other, potentially affecting systemic health and underscoring their interconnectedness. This mutual relationship highlights the importance of considering both systems together when studying and addressing various health conditions.

In this review, we summarize the current knowledge of the oral-gut axis, including the role of the circulatory system in bacterial translocation and systemic inflammation while highlighting the importance of the interconnectedness of these systems. Both oral pathogens, *P. gingivalis* and *F. nucleatum* have been implicated in the pathogenesis of colon cancer by directly affecting the host cell signaling pathways involved in cell proliferation, apoptosis, and DNA damage repair. These microbial interactions can further exacerbate inflammation and promote the growth and survival of colorectal cancer cells. The presence of these oral pathogens in the gut underscores the connection between of the oral-gut axis in the context of carcinogenesis.

## The immune response in oral and intestinal mucosa

The mucosal immune system constitutes a specialized and region-specific defense network safeguarding a substantial portion of the inner surface of human anatomy, encompassing the mucosal linings of the respiratory system, urogenital tract, oropharyngeal region, GI tract, and also the exocrine glands. Specialized functions in the oral cavity and GI tract lead to variations in immune responses. These include differences in mucosal surfaces, microbial composition, and immune cell populations. The main roles of the oral cavity, digestion and speech articulation, require different immune adaptations compared to the GI tract, which primarily handles nutrient absorption and hosts a larger and more diverse microbial community. These distinctions contribute to unique immune profiles and responses in each region (Moutsopoulos and Konkel, 2018).

The intricate mucosal surfaces of the oral cavity, including the gingiva, tongue, and buccal mucosa, constitute the first line of defense against pathogens. The oral mucosa components ensure the integrity of oral tissues and form a complex defense network that protect against potential threats while maintaining tolerance to harmless entities. Oral epithelia are multilayered barriers with highly diverse antigenic responses and different expression patterns of cytokeratin. Salivary glands secrete saliva that contains enzymes and proteins, like lysozyme and lactoferrin, which possess antimicrobial properties (Moutsopoulos and Konkel, 2018). The GI immune system has a unique characteristic known as oral tolerance allowing the immune system to be unresponsive or tolerant to ingested protein antigens. Although the immune system becomes more mature and less flexible as we age, the oral cavity and gut immune systems still actively maintain tolerance to dietary antigens in adulthood.

Both oral and intestinal mucosa have specialized lymphoid tissues known as mucosa-associated lymphoid tissue (MALT). In the oral cavity, MALT includes tonsils and adenoids, while in the gut, it encompasses Peyer’s patches, isolated lymphoid follicles. These lymphoid tissues contain immune cells, including T cells, B cells, innate lymphoid cells (ILCs), and antigen-presenting cells (APC), which play a crucial role in immune responses (Moutsopoulos and Konkel, 2018; Suárez et al., 2021). In the gut, innate immune tactics involve employing a combination of defenses such as a protective mucus layer, antimicrobial peptides (AMPs), and the coordinated action of ILCs. These mechanisms work together to contain a significant portion of the microbial community within the interior space of the intestinal tract. ILCs are innate equivalents of T cells without antigen-specific receptors, playing pivotal roles in immune responses by producing effector cytokines, maintenance of mucosal barriers, such as the epithelial lining of the oral and intestinal mucosa, and regulating other immune cells including T cells, B cells, and dendritic cells. They are present in lymphoid and non-lymphoid organs, along with mucosal barriers exposed to allergens, commensal microbes, and pathogens whereas these cells are uncommon in the bloodstream. Different ILC subsets, including ILC1s, ILC2s, and ILC3s, each have their own specific functions and cytokine profiles. ILC3s are abundant in the intestinal mucosa and have been implicated in inflammatory bowel disease (IBD). They play a dual role: promoting tissue repair and maintaining mucosal homeostasis, while also contributing to inflammation when dysregulated. Additionally, ILCs have been suggested to have roles in tumor immune surveillance, contributing to the recognition and control of tumor cells. However, their specific functions and contributions to antitumor immunity are still being studied (Panda and Colonna, 2019).

Regulatory T cells (Tregs) and other regulatory immune cells play an essential role in suppressing immune responses and maintaining tolerance (Upadhyay et al., 2013). Microbial-associated molecular patterns (MAMPs) are conserved structural components of microorganisms that are recognized by the innate immune system. Biofilm and bacterial metabolic products like lipopolysaccharides (LPS) are the main MAMPs that can stimulate the expression and production of pro-inflammatory cytokines through activation of toll-like receptors (TLRs). TLRs serve as crucial mediators in the inflammatory pathways, significantly contributing to the orchestration of immune responses against a diverse range of ligands originating from pathogens. They play a vital link connecting adaptive immunity with innate immunity. TLRs recognize MAMPs and activate signaling pathways that lead to the production of pro-inflammatory cytokines and chemokines, as well as the upregulation of co-stimulatory molecules and antigen-presenting molecules (Mackey and McFall, 2006).

Moreover, nucleotide-binding oligomerization domain-like receptors (NLRs) also communicate with several innate immune sensors and receptors in the oral and intestinal epithelial cells. NLRs binding and downstream signaling by cytokines, chemokines, and antimicrobial peptides production provoke an inflammatory reaction (Franchi et al., 2009; Cekici et al., 2014). One major function of NLR proteins is to regulate and modulate inflammatory signaling pathways, including NFκ-B and MAPK. Once activated, NFκ-B translocates to the nucleus, where it binds to target genes and induces the transcription of pro-inflammatory cytokines and other immune response genes. Similarly, the activation of MAPK signaling pathway leads to the production of pro-inflammatory cytokines, including tumor necrosis factor alpha (TNF-α), interleukin-6 (IL-6) and interleukin-1 beta (IL-1β) (Franchi et al., 2009). Therefore, NLR proteins play an important role in maintaining the balance between immune defense and inflammatory damage in the oral and gut microenvironment.

Neutrophils play a vital role in homeostatic immunity by serving as the front-line defenders against microbial threats. Rapidly responding to infection signals, neutrophils migrate to affected areas, where they engulf and neutralize pathogens through phagocytosis and releasing antimicrobial proteins and cytokines modulating the behavior of other immune cells. Additionally, neutrophils support the epithelial barrier's function by participating in the tissue repair process (Moutsopoulos and Konkel, 2018).

Dendritic cells (DCs) are another important antigen-presenting cell that play a critical role in linking innate and adaptive immunity. They derive from hematopoietic stem and progenitor cells (HSPCs) in the bone marrow and are found in various locations throughout the body. Being the most potent cells responsible for activating and directing naïve T cells, DCs in the oral region hold significant importance in orchestrating both immune responses and tolerance within the oral mucosa (Hovav, 2014). Unlike in murine models, the comprehensive examination of dendritic cells in human oral tissues remains limited, with the primary focus typically centered on Langerhans cells (LCs) (Hovav, 2014). Nonkeratinized mucosal regions, including the soft palate, ventral tongue, lip, and floor of the mouth, show the highest LC concentration, while the keratinized mucosa of the hard palate displays the lowest LC density (Daniels, 1984). LCs within the oral mucosa effectively collect oral fluids and bacteria, with their dendritic extensions reaching towards the surface, often constituting a diverse group. LCs become mobile and mature in response to inflammatory cytokines and pathogen-associated molecular patterns (PAMPs) released by oral mucosal pathogens. LCs are primarily responsible for presenting exogenous antigen-derived peptides through major histocompatibility complex (MHC) class II presentation to CD4+ helper T-cells and MHC class I-restricted cytotoxic (CD8+) T-cell responses. Furthermore, LCs stimulate Natural killer (NK) cells by producing cytokines, including IL-12 (Hovav, 2014).

Activated DCs migrate to the GALT, where they interact with naïve T cells, thereby initiating the adaptive immune response. This migration and subsequent T cell activation are crucial for immune surveillance and responses in diverse body regions (Hovav, 2014). A deeper exploration is necessary to comprehend how DC activation in oral epithelia and their migration across different tissues contribute to coordinating immune responses and maintaining immune tolerance throughout the body.

Th17 cells, a subset of CD4+ T cells, specialize in orchestrating immune responses at mucosal surfaces by producing IL-17 and other cytokines that recruit neutrophils and enhance antimicrobial defenses. The coordinated efforts of neutrophils and Th17 cells illustrate the intricate collaboration between innate and adaptive immunity in safeguarding mucosal homeostasis and combating infections. This dynamic interaction plays a crucial role in maintaining the delicate balance between protective immunity and immune tolerance within mucosal environments (Suárez et al., 2021).

The intestinal epithelium is acknowledged as the principal axis of mucosal immunity, given that approximately 70% of the total lymphocyte population resides within the gastrointestinal tract (Suárez et al., 2021). The GI mucosal immune system comprises three principal components: the epithelial layer, lamina propria, and the MALT, known as gut-associated lymphoid tissue (GALT). The epithelium and lamina propria serve as the frontline defenses, while the GALT functions as the central hub where adaptive immune responses are instigated, specifically within the context of the GI tract (Wu et al., 2014). The epithelium layer is primarily composed of intestinal epithelial cells (IECs), organized into distinct structures known as villi. Other specialized cell types coexist, including Goblet cells, tuft cells, enteroendocrine cells, and M cells. Goblet cells lubricate and shield the intestinal epithelial surface by secreting mucins. (Suárez et al., 2021). Control of paracellular permeability is crucial for preventing microbial invasion, and this is achieved through the regulation of various types of intercellular junctions (Suárez et al., 2021). The gut-associated lymphoid tissue consists of Peyer’s patches and isolated lymphoid follicles (Wu et al., 2014). Goblet cells also play a role as luminal antigen-presenting cells to CD103+ DCs, facilitating the differentiation of Tregs. DCs extend their dendrites into the epithelium to capture antigens and subsequently migrate to the lamina propria, subsequently draining to secondary lymphoid tissues. Meanwhile, specialized M cells, present in the epithelium of Peyer’s patches, facilitate the transfer of antigens to DCs, macrophages, and other APCs. In secondary lymphoid tissues, naïve T cells undergo activation upon interaction with APCs and subsequently migrate to the lamina propria (Wu et al., 2014).

Overall, the oral and gut immune responses exhibit both similarities and differences, sharing common features in terms of lymphoid tissues and immune cell populations. On the other hand, microbial compositions, exposure to antigens, immune tolerance mechanisms, and antibody production are different. It has been reported that immune cells present in the oral draining lymph nodes can transmigrate to various other lymphoid organs, including the gut (Morton et al., 2014). Consequently, oral inflammation could lead to the emergence of T cells reactive to oral pathobionts. These T cells have the potential to migrate from the oral mucosa to the intestine, where they could become activated by specific microbes, potentially leading to the onset of intestinal inflammation.

Furthermore, the gut mucosa employs alternative immune mechanisms to regulate the production of microbiota-responsive effector T cells. Within the gut, a specific group of ILCs expressing MHC class II molecules hinders the expansion of T cells specific to the microbiota. It is plausible that the oral mucosa and gingiva do not possess these intricate tolerogenic mechanisms observed in the gut. Therefore, acute inflammation in the oral mucosa might lead to the development of microbiota-responsive T cells with potential pathogenic characteristics. Th17 cells normally present in the gut do not induce disease, unlike their significant pathogenic role in promoting inflammation driven by commensal bacteria in the oral cavity in both mice and humans. Upon migration to the gut mucosa, these cells may undergo functional conversion into Th17/Th1 mixed phenotype cells capable of producing interferon-γ (IFN-γ). This conversion might contribute to the development of colitis triggered by oral-origin T cells. In summary, migratory Th17 cells represent a double microbiota and immune mechanism linking the oral and gut environments (Kitamoto et al., 2020).

A comprehensive understanding of the immune dynamics operating in these sites is crucial for unraveling the complex interplay between the oral and gut immune responses. Such insights could pave the way for novel strategies aimed at modulating immune reactions and designing interventions for diseases that involve both oral and gut components.

## Dysbiosis in the oral microbiota and host response

The oralome comprises the dynamic interactions coordinated between the ecological community of oral microorganisms and the host within the oral cavity (Radaic and Kapila, 2021). The mouth harbors over 700 species of bacteria, as well as fungi, viruses, and protozoa, making it the second-largest and diverse microbiota after the gut (Peterson et al., 2009). Interindividual differences also exist, but the principal function of the microbiome is the same in every person. Oral commensal microorganisms help maintain microbiota balance, inhibiting pathogen attachment and invasion. They support oral health by competing for resources, producing antimicrobials, and modulating the host immune response (Wade, 2013). The oral microbiome has been extensively characterized using both cultivation and culture-independent molecular techniques, such as 16S rRNA cloning. The six most abundant phyla identified in the oral microbiome are: Firmicutes, Bacteroidetes, Proteobacteria, Actinobacteria, Spirochaetes, and Fusobacteria, which together comprise 96% of the taxa (Dewhirst et al., 2010).

Biofilms are organized communities of similar or different aggregated bacterial cells encased in a self-produced polymeric matrix adhered to a surface (Donlan, 2002). Oral biofilm formation begins with the initial attachment of planktonic “early colonizer” bacteria, primarily saccharolytic aerobes and facultative anaerobes that utilize glycoproteins and salivary mucins as nutrients. Streptococcus species, making up around 80%, dominate this early colonization phase (Kreth et al., 2009). Following the attachment, surface-bound bacteria undergo shifts in their metabolic and gene expression profiles resulting in the generation of extracellular polymeric substances, including polysaccharides, proteins, lipids, and extracellular DNA (Radaic and Kapila, 2021). *F. nucleatum* plays a key role as a “bridge” bacterium within the biofilm by adhering to a diverse array of co-aggregated species through surface adhesins (Coppenhagen-Glazer et al., 2015).

The oral biofilm presents varying levels of oxygenation across its structural composition, providing the necessary environmental conditions for the attachment and proliferation of proteolytic obligate anaerobes, commonly referred to as “late colonizers” (Wade, 2013). Ahn et al. (Ahn et al., 2016) examined how *F. nucleatum* and *P. gingivalis* infections affect reactive oxygen species (ROS) production and bacterial complex formation in human oral cells. Despite both being anaerobic bacteria, *F. nucleatum* and *P. gingivalis* have different oxygen tolerances but are commonly found together at infection sites. ROS production primarily relies on NADPH oxidases (NOX) enzymes. Notably, *F. nucleatum* exhibits a 20-fold higher NOX activity compared to *P. gingivalis*, giving it a much greater ability to utilize oxygen molecules (Diaz et al., 2002). Similarly, the presence of *F. nucleatum* increases *P. gingivalis* attachment to human gingival fibroblasts by a factor of ten. Ultimately, the study (Ahn et al., 2016) revealed that *F. nucleatum* contributes to the co-aggregation of *P. gingivali*s, promoting the formation of bacterial complexes.

The most prevalent oral diseases that are dental caries and periodontitis are both associated with a disruption in the balance of oral microbiota. Dental caries is a biofilm disease caused by multiple microorganisms, influenced by dietary habits leading to the demineralization of tooth enamel through acid production by oral pathogens fueled by dietary sugars (Bowen et al., 2018). Periodontitis is a chronic inflammatory disease initiated by a dysbiotic biofilm in the gingival pocket. Its polymicrobial nature is driven by variations in subgingival microbiota composition and interactions, rather than new bacterial colonization (Aas et al., 2005; Bowen et al., 2018). The subgingival microbiota is typically dominated by *Firmicutes*, with fewer *Actinobacteria* and *Bacteroidetes*. In contrast, periodontitis is characterized by the presence of *P. gingivalis*, *Tannerella forsythia (T. forsythia*), *Treponema denticola (T. denticola*), and *F. nucleatum* (Paster et al., 2001; Donlan, 2002). Dominant species in the subgingival biofilm play a crucial role in driving dysbiosis and inflammatory response, in periodontitis development (Aas et al., 2005).

The aggregation of bacteria triggers local inflammation, increasing crevicular gingival fluid flow, leading to bleeding, and providing protein-rich nutrients. This promotes the growth of the Gram-negative anaerobes (Hoare et al., 2019). Within the periodontal pocket, the initial host response to the dysbiotic subgingival community involves NK cells, neutrophils, and granulocytes, initiating early inflammation. Subsequently, lymphocytes infiltrate, facilitating antigen presentation to dendritic cells. T cells, including CD8+ and CD4+ cells, generate a proinflammatory environment rich in cytokines like TNF-α, IL-1, IL-4, IL-10, IFN-γ, and transforming growth factor β (TGF-β) (Pihlstrom et al., 2005). This inflammatory cascade leads to changes in the subgingival environment, contributing to shifts in the subgingival biofilm composition that drive the progression of periodontitis. Interestingly, certain periodontal pathogens directly play a role in promoting chronic inflammation by activating specific intracellular pathways (Hoare et al., 2019).

**Figure 1:**
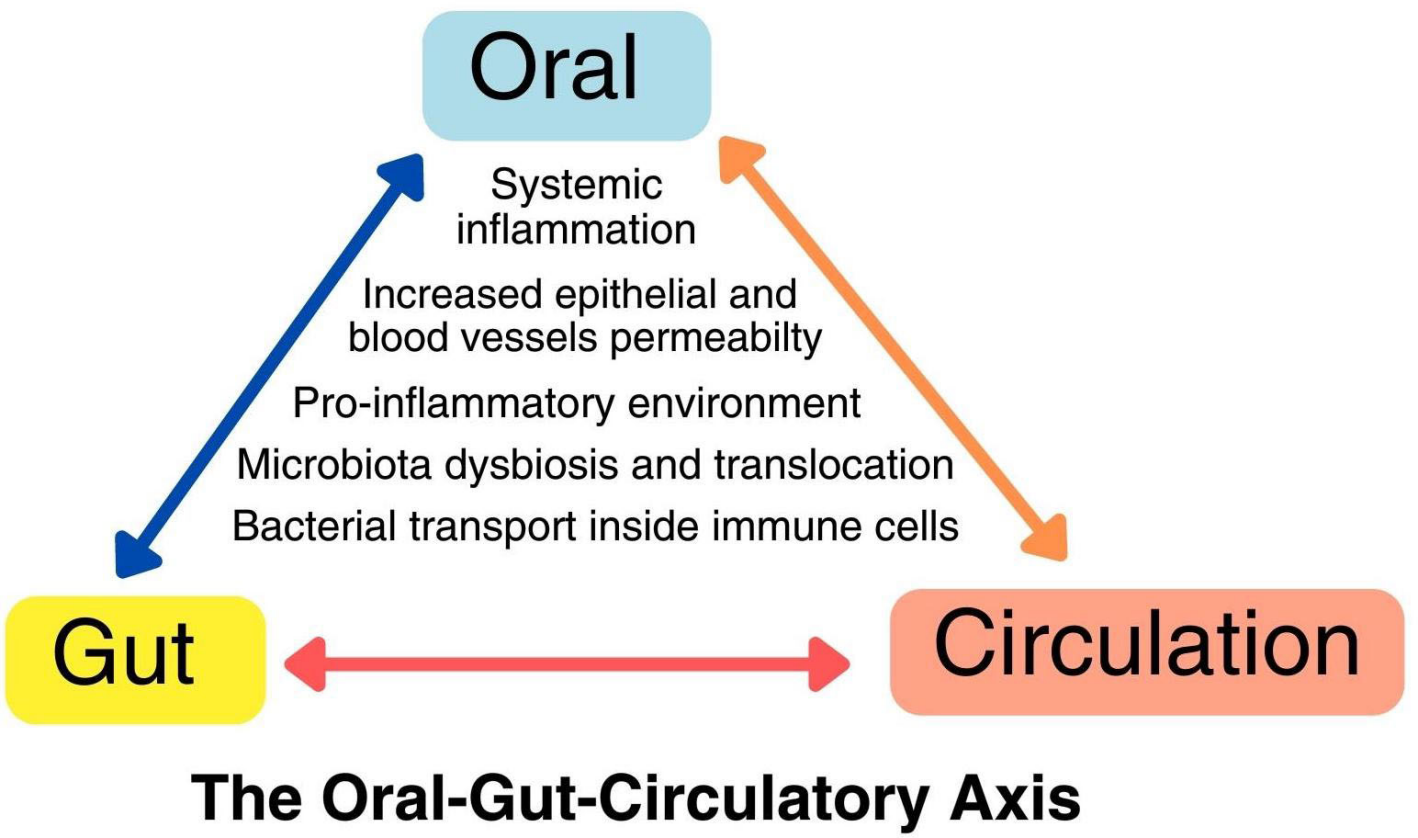
The oral-gut-circulatory axis. This axis involves a pro-inflammatory state and the recruitment of immune cells due to local dysbiosis, resulting in heightened permeability in both epithelial cells and endothelial cells of blood vessels. This increased permeability allows pathogens to spread through transient bacteremia and intracellular transport within immune cells like macrophages and neutrophils. This axis operates in a dynamic, bidirectional manner, leading to direct and indirect changes at distant body sites.

Several studies have linked periodontitis to systemic diseases, such as cardiovascular, diabetes mellitus, respiratory disease, adverse pregnancy outcomes (Geisinger et al., 2016), and increased risk of pancreatic and colon cancers (Pihlstrom et al., 2005; Momen-heravi et al., 2018; Kim et al., 2019; Koliarakis et al., 2019). Nevertheless, the available evidence about remains limited, necessitating further research to elucidate the nature of this connection and the underlying mechanisms. Potential mechanisms linking oral infections to systemic conditions encompass the spread of oral pathogens through transient bacteremia and intracellular infection in immune cells, the release of oral microbial toxins into the circulation at the site of injury, and interactions between oral pathogens and the host’s immune response, resulting in systemic inflammation (Xiaojing et al., 2000). Further prospective investigations are imperative to gain a comprehensive understanding of the causal mechanisms linking oral dysbiosis and its potential impact on the development of colon cancer (**Figure 1**).

## The circulation as a key component in the oral-gut axis

The mechanisms underlying bacterial leakage into the bloodstream remain unknown, with hypotheses ranging from dendritic cell processes, and oral pathogen-immune cells transport to dysfunctional epithelial junctions. Some studies reported pathogen *P. gingivalis* in the bloodstream of healthy individuals and those with periodontitis after activities like tooth brushing, flossing, or chewing food (Horliana et al., 2014). Tsukasaki et al. observed the formation of oral bacterial colonies in liver and spleen cells following prolonged ligature placement around a tooth in a murine model of periodontitis. The bacterial species were also present in the oral cavity but were notably absent in fecal samples. This suggests that the systemic spread of oral bacteria occurs when the oral barrier is compromised (Tsukasaki et al., 2018).

Severe periodontitis is associated with elevated levels of pro-inflammatory mediators and recruitment of immune cells to the affected site, including increased neutrophil numbers in the blood. The inflammatory response caused by this inflammatory chronic disease can disrupt the tight junctions between endothelial cells, leading to increased permeability of blood vessels. This increased permeability allows inflammatory mediators, bacterial components, and immune cells to enter the vessel walls and surrounding tissues, promoting systemic inflammation (Nakajima et al., 2010). Periodontal pockets surface area presents a large bacterial biofilm accumulation, ranging from 50 cm^2^ to 200 cm^2^, allowing bacterial products such as lipopolysaccharides or proteases to diffuse into the blood stream (Hujoel et al., 2001).

Via transient bacteremia, bacterial infection is cleared by the immune system. However, some can evade the immune response and survive in the bloodstream. It is known that leukocytes are not effective in recognizing and engulfing bacteria in high-velocity liquids, although erythrocytes can attract bacteria by electrical charges on their surface and kill them by oxidative attack. Despite this, some bacteria can survive in the liver or spleen promoting low-grade inflammatory response and inducing cytokine secretion by other tissues/organs (Minasyan, 2014). Bacterial reservoir inside erythrocytes provides a long-term bacteria survival causing antibiotics ineffectiveness and immune reactions (Minasyan, 2017).

Individuals with periodontitis have elevated circulatory levels of pro-inflammatory cytokines, such as IL-1, IL-6, and TNF-alpha. Once in the circulation, these cytokines can then exert their effects on other tissues and organs, contributing to systemic inflammation and potentially leading to the development of comorbidities at distant sites (Nakajima et al., 2010). In addition, these cytokines are released in response to bacterial pathogens in the gums, acting as immune cells recruiters at the infection site.

Recent research suggests a potential link between immune cells activated in the oral cavity and the development of gut inflammation. It proposes that these immune cells can migrate from the oral cavity to the gut, where they can play a role in promoting inflammation. This is referred to as an indirect pathway, as the immune cells themselves do not directly trigger gut inflammation. Instead, they contribute to the inflammatory process by priming other cells or interacting with various components of the immune system. An alternative idea, called the Trojan Horse hypothesis, suggests that immune cells could transport pathogens throughout the body.

In periodontitis, *P. gingivalisis* found intracellularly in DCs, which affects the differentiation of these immune cells while using them as vehicles to enter the circulation and disseminate to distant organs (Carrion et al., 2012). On the other hand, recent research has shown that human neutrophils can carry and spread viable *F. nucleatum* after phagocytosis by using both an *in vitro* microfluidic device and a zebrafish model. The researchers previously found that *F. nucleatum* can suppress immune responses in neutrophils and survive within them, suggesting that intracellular bacterium can avoid the host’s immune defenses and spread from the oral cavity (Ellett et al., 2023). Additionally, macrophages have been proposed as a communication network linking the oral cavity to distant sites. *P. gingivalis* has been shown to invade and persist within resident macrophages. Moreover, studies in animal models indicate that *P. gingivalis*-infected macrophages can produce cytotoxic extracellular vesicles, damaging distant organs. *P. gingivalis* has evolved intricate strategies to evade macrophage antimicrobial defenses. Understanding *P. gingivalis*-macrophage interactions may yield insights into disease mechanisms and potential therapies (Lin et al., 2022). Moreover, *F. nucleatum* can also survive within the cytoplasm of infected macrophages by the expression of indoleamine-2,3-dioxygenase, which can amplify the impairment of peripheral blood lymphocyte function. This dual effect allows *F. nucleatum*-infected macrophages to evade cellular death (Xue et al., 2018). These mechanisms suggest that common oral bacteria could connect oral and systemic diseases by utilizing the body’s immune cells for transport. Further investigation should be done to study if this mechanism for immune cells-mediated bacterial dissemination could apply to other oral bacterial species and potentially contribute to the link between oral and systemic diseases.

## From oral bacterial translocation to gut microbiota dysbiosis

**Figure 2:**
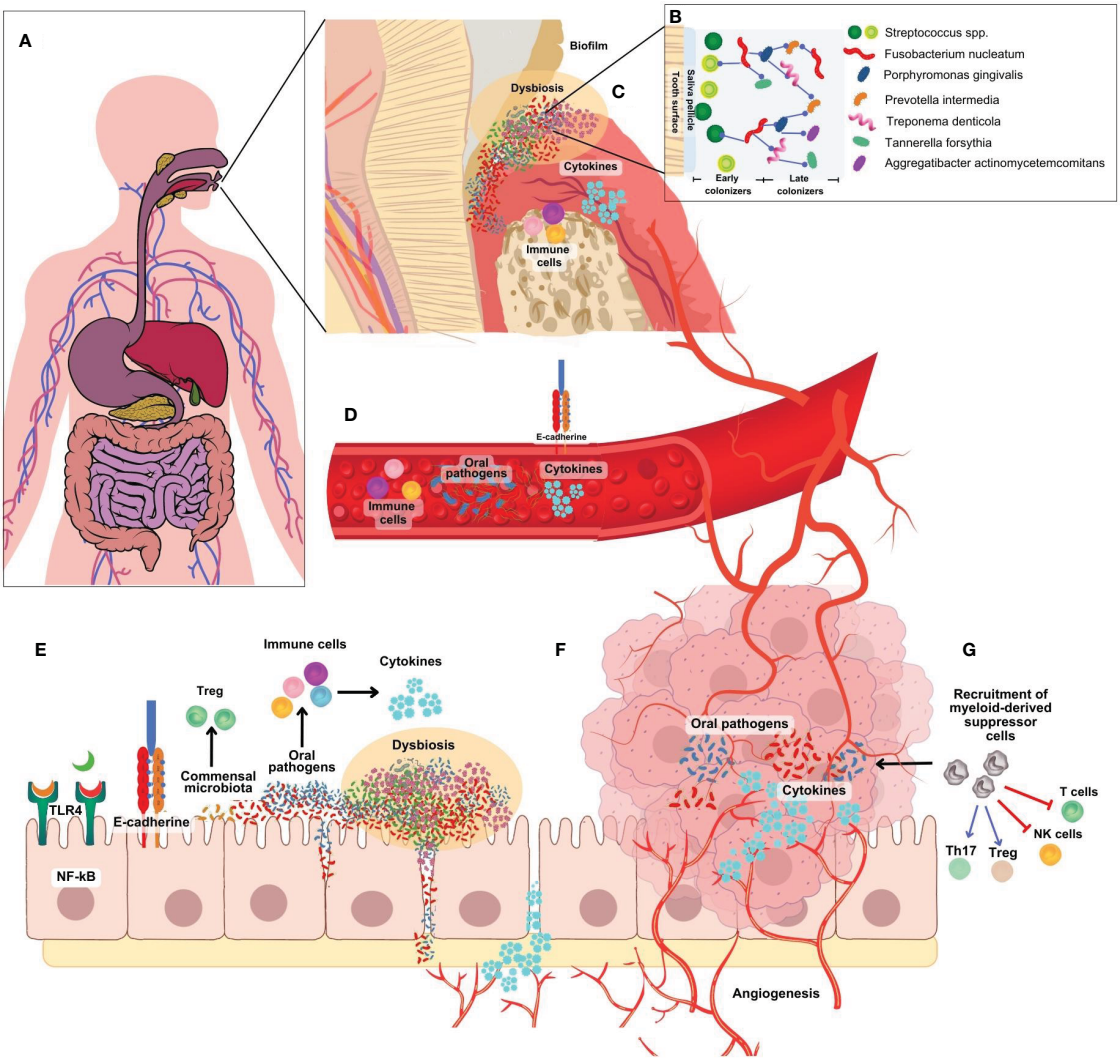
**(A)** Microbiome research has traditionally focused on specific organs, neglecting the continuous anatomical connection between the mouth and gut. **(B)** The development of an *F nucleatum-P. gingivalis* bacterial complex is central to the formation of oral biofilm. *F nucleatum* acts as a bridge attaching early colonizers like *Streptococcus* spp, *Actinomyces* spp, *P. gingivalis* and others. **(C)** In the periodontal pocket, initial host response is characterized by the infiltration of NK cells, neutrophils, granulocytes, and lymphocytes to present antigens to dendritic cells. T cells secrete cytokines such as TNF- α, IL-1, IL-4, IL-10, IFN- γ, TGF- β, RANK-L. **(D)** Bacteria, metabolites and inflammatory factors can travel from the oral cavity to other parts of the body into the blood vessels by increased endothelial permeability. **(E)** Oral bacteria, particularly *F nucleatum* and *P. gingivalis*, can infiltrate intestinal cells, provoke pro-inflammatory cytokine production, and induce a pro-inflammatory environment **(F)**. They activate various pathways linked to inflammation and cancer development and can disrupt the colon’s epithelial barrier, increasing permeability to opportunistic bacteria and promoting chronic inflammation, angiogenesis, and cancer progression. **(G)** A diverse array of immune cells are recruited to the tumor site, including myeloid-derived suppressor cells (MDSCs). MDSC block T cells and NK cells activation, and recruitment and activation host suppressor cells such as FoxP3+ regulatory T cells.

The study of the oral and gut microbiomes has mostly been conducted in an organ-specific manner, which overlooks the fact that the mouth and gut are anatomically continuous regions; moreover, both regions are chemically connected through the passage of salivary fluids and digested food through the GI tract (**Figure 2A**). Saliva is estimated to contain about 10^6^ bacteria per ml, which would result in people orally ingesting as many as 10^12^-10^13^ bacteria day (Von Troil-Lindén et al., 1995). The oral cavity is the entry point for the digestive tract and is continuously exposed to various external factors, including microorganisms, nutrients, and xenobiotics. The Human Microbiome Project (Peterson et al., 2009) showed that more than half of the total bacteria in the human body are present in the GI tract (29%) and the oral cavity (26%). Interestingly, oral bacteria have also been found in distant sites such as the pancreas and gut, indicating direct crosstalk between microbiota at different locations (Chung et al., 2021).

The transmission and invasion of oral pathogens in the gut require the presence of at least two essential factors, one within the oral microbiota and the other within the gut microbiota. The first prerequisite is an elevated number of oral bacteria in the mouth caused by oral dysbiosis which enhances the likelihood of gut translocation. The second prerequisite requires the disruption of the intestinal mechanisms to resist colonization by opportunistic bacteria, which is typically conferred by gut dysbiosis. This disruption may be a necessary step to allow oral pathobionts, which have successfully traversed the gastric barrier, to establish colonization within the gut (Kitamoto and Kamada, 2022). Kageyama et al. (Kageyama et al., 2023) investigated both saliva and stool samples using 16S rRNA gene amplicon analysis and the amplicon sequence variant (ASV) approach to explore the translocation of oral bacteria to the gut. The study found that ASVs shared with an individual’s salivary microbiota were present in the gut microbiota of 72.9% of subjects. Notably, the sharing and similarity of oral-gut microbes were more pronounced within an individual than across different individuals (Schmidt et al., 2019).

Overall, the acidic environment of the stomach plays an important role in protecting the host against potential infections from ingested microbes, as well as in regulating the composition of the gut microbiota. Research findings indicate that over 99% of oral microbes that are ingested are rendered inactive or eliminated as they traverse the stomach. The high acidity of the stomach environment makes it inhospitable for the survival of most bacteria. However, some microorganisms, such as *Helicobacter pylori (H. pylori)*, are adapted to survive in the acidic environment of the stomach and can colonize the gastric mucosa (Hunt et al., 2015). Long-term colonization by *H. pylori* can cause achlorhydria and decreased acid secretion, leading to changes in the gastric microbiota (Hunt et al., 2015). Additionally, gut colonization by oral-origin bacteria, Streptococcus and Veillonella, is observed in patients with gastric achlorhydria caused by long-term use of proton pump inhibitors (Schmidt et al., 2019). Furthermore, aging displayed a significant correlation with an increased abundance of oral bacteria in the gut microbiota. This association could be attributed to the diminished barrier function of the gastrointestinal tract due to aging-related alterations (Kageyama et al., 2023).

The gut microbiota contains 1000-1150 bacterial species, some of the most abundant groups are *Bacteroidetes, Dorea/Eubacterium/Ruminococcus, Bifidobacteria, Proteobacteria, and Streptococci/Lactobacilli* (Qin et al., 2010). Maintaining a balanced relationship between the host and commensal microorganisms is crucial for proper functioning. The host provides a fit environment and nutrients for the microorganisms to thrive, while the commensal bacteria contribute to various physiological functions and offer protection against pathogens invasion. This intricate balance is achieved through complex interactions between the host and microbiota (Lee et al., 2013). Oral administration of *P. gingivalis* in C57BL/6 mice alters gut microbiota with an increase in *Bacteroides* and *Staphylococcus* and a decrease in commensal species such as *Firmicutes* and *Lactobacillus* when compared to sham-inoculated mice (Nakajima et al., 2015). *Lactobacillus* species are recognized as probiotics, known for their ability to promote digestive health and support immune function. Decreased levels of probiotic bacteria can disrupt the gut balance, leading to dysbiosis and compromised intestinal barrier. This may increase intestinal permeability, allowing toxins and bacteria to enter the bloodstream and potentially trigger inflammation and health problems (Arimatsu et al., 2014; Gao et al., 2015; Kobayashi et al., 2020).

In mice administered with *P. gingivalis*, the expression of genes responsible for intestinal alkaline phosphatase (Akp3) and the tight junction protein (Tjp1) in the small intestine was both decreased. (Arimatsu et al., 2014). Additionally, Kobayashi et al. (Kobayashi et al., 2020) found that the effects on the gut microbiota were not solely due to *P. gingivalis*, but also due to the other oral bacteria such as *Streptococcus mitis, Streptococcus salivarius, Porphyromonas nigrescens*. Interestingly, the study found that dysbiosis led to a decrease in the abundance of Th17 cells, a type of immune cell involved in protecting against pathogens, and a decrease in the production of immunoglobulin A (IgA), an antibody important for mucosal immunity in the small intestine. IgA and Th17 cells play important roles in maintaining gut homeostasis and protecting against pathogen invasion. With the growing body of evidence highlighting the significance of interactions between gut microbiota and immune cells in maintaining the immune system balance, Morton et al, utilizing Kaede transgenic mice, observed a bidirectional immune cell trafficking between the gut and other organs (Morton et al., 2014). The migration of oral immune cells to the gut is a pivotal aspect of the mouth-gut axis in intestinal inflammatory conditions, such as colitis. During periodontal inflammation, Th17 cells are generated in the oral draining lymph nodes, specifically recognizing oral bacteria. Upon reaching the gut, these orally primed Th17 cells can be activated by translocated oral pathobionts, contributing to colitis development (Kitamoto and Kamada, 2022).

Interestingly, Nagao et al. (Nagao et al., 2022) hypothesized that oral pathogens in the oral cavity, known as pathobionts, could migrate to the intestinal tract and trigger an immune response that exacerbates periodontitis. They infected mice with *P. gingivalis* showing that the infection provoked pathobiont migration from the oral cavity to the intestine where they were recognized by DCs triggering the Th17-type immune response, leading to the production of IL-17 and other pro-inflammatory cytokines. Interestingly, the study revealed that Th17 cells can migrate from the intestine to the oral cavity upon oral infection. This suggests that the migration of Th17 cells may be one of the mechanisms by which the gut microbiota can affect periodontitis. These findings suggest that the oral-gut axis is bidirectional, and that the gut microbiota can affect the homeostasis of the mouth. It is crucial to further explore and understand the specific mechanisms involved in the communication between the oral and gut microbiota to gain insights into their collective impact on oral, gut, and systemic health. The examination of host-microbe interactions in murine models has played a crucial role in unraveling the gut-axis relationship. However, given the variations between the microbiotas of mice and humans, it is imperative to invest additional effort into comprehending how these findings apply to humans (Kitamoto and Kamada, 2022).

An imbalance in the oral microbiome can not only lead to oral disorders such as dental caries and periodontitis, but also systemic diseases such as irritable bowel syndrome, IBD (Tsuzuno et al., 2021; Kitamoto and Kamada, 2022), and colorectal cancer (CRC) (Flemer et al., 2018; Tortora et al., 2022). Metagenomic sequencing analysis revealed the presence of four species enriched in tumor samples in CRC patients with oral cavity origin: *Porphyromonas asaccharolytica, F. nucleatum, Prevotella intermedia*, and *Parvimonas micra*. Remarkably, these bacteria were found to form mutually beneficial networks within the microbial community where *F. nucleatum* occupied a central position within this network, suggesting a pivotal role for oral bacteria in shaping these interactions (Dai et al., 2018) similar to the role that *F. nucleatum* plays in oral biofilm as a “bridge” bacteria between early and late colonizers. Bacterial biofilms that invade the mucus layer can be observed on the colon mucosa in around 50% of CRC patients and roughly 13% of individuals without the disease (Tomkovich et al., 2019). These findings suggest that the composition and arrangement of the microbiota, rather than the overall health status of the human donor, are connected to the development of tumors. Additionally, given the association between bacterial biofilms and CRC, it is crucial to gain insight into the functional significance of bacterial organization within the colon mucosa in relation to CRC. The next phases of research should involve the retrieval of oral and colonic mucosal biofilms for comprehensive analyses, focusing on bacterial community composition and functional profiling to identify commonalities shared among these microbiota, providing valuable insights into the mechanisms underlying their role in disease development.

## Oral pathogens in the carcinogenesis of colon cancer

The development and progression of colon cancer is associated with several factors including genetics, environmental, diet, lifestyle, and microbiota (Tortora et al., 2022). Several studies have highlighted the relationship between the gut microbiota and colon cancer (Cho et al., 2014; Fulbright et al., 2017; Koliarakis et al., 2019; Tortora et al., 2022). Oral diseases, such as periodontitis, involve chronic inflammation triggered by a multispecies bacterial community in the subgingival region in the mouth. While inflammation mainly occurs in the oral cavity, studies reveal that inflammatory agents, subgingival bacteria, and their components can disseminate, contributing to extraoral diseases like cancer (Hoare et al., 2019; Koliarakis et al., 2019). The association between periodontal disease and CRC was established in the Nurses’ Health Study, in which women with moderate or severe periodontitis were at moderately increased risk of developing CRC (Momen-heravi et al., 2018; Nwizu et al., 2020).

As previously mentioned, the formation of an *F. nucleatum-P. gingivalis* bacterial complex is central to the pathogenesis of periodontitis (Ahn et al., 2016). *F. nucleatum* acts as a bridge attaching other early colonizers like *Streptococcus* spp, *Actinomyce*s spp, *P. gingivalis* and others (**Figure 2B** *).* In the periodontal pocket (**Figure 2C**), the initial host response is marked by the infiltration of NK cells, neutrophils, and DCs, followed by the subsequent influx of lymphocytes after antigen presentation. Recruited T cells subsequently contribute to the response by secreting cytokines like TNF-α, IL-1, IL-4, IL-10, IFN-γ, TGF-β, and receptor activator of nuclear factor kappa-B ligand (RANK-L) (Hoare et al., 2019). One of the complications of periodontal disease is the migration of bacteria from the oral cavity to other parts of the body. This occurs because there is an increase in the number of oral bacteria in the sub-gingival biofilm in intimate contact with the ulcerated gingiva creating an entry point for oral bacteria into the bloodstream, facilitating their dissemination to remote locations such as the colon (**Figure 2D**) (Nwizu et al., 2020). These bacteria are mostly multispecies with high numbers of *Clostridium, Peptostreptoccocus* and *Fusobacterium*.(Hoare et al., 2019) In the gut (**Figures 2E-G**), oral bacteria, can invade and adhere to intestinal epithelial cells, increase the production of pro-inflammatory cytokines, and contribute to a pro-inflammatory microenvironment (Bashir et al., 2016). These findings imply that *F. nucleatum* may exert a significant indirect influence on the development of periodontal diseases by promoting the proliferation of *P. gingivalis*, potentially contributing to the etiological factors involved. The ability of *F. nucleatum* to adhere to both host cells and other bacterial species enhances its capacity to form complex microbial communities and promote the colonization of harmful bacteria in the gut. These findings highlight the interconnected nature of the oral-gut axis and suggest that *F. nucleatum* and *P. gingivalis* may have significant influence not only in the oral cavity but also in gut (**Figure 2F**) (Castellarin et al., 2012). Interestingly, *F. nucleatum* and *P. gingivalis* are known to synergistically promote oral cancer progression (Binder Gallimidi et al., 2015). Similar studies should be proposed to study potential synergy between both oral pathogens and gut dysbiosis in colon cancer.

In the study by (Tsuzuno et al., 2021) *P. gingivalis* amplified gastrointestinal inflammation by directly engaging with the intestinal epithelial barrier in a susceptible host and showed a higher colitogenic potential compared with other periodontal pathogens such as *Prevotella intermedia* and *F. nucleatum* in mice. In summary, the potential mechanisms through which *P. gingivalis* reduces the protein level of ZO-1 *in vivo*, also known as tight junction protein-1, encompass several interconnected events: detachment of intestinal mucus bacterial invasion into intestinal epithelial cells, and cytosolic degradation of ZO-1(Tsuzuno et al., 2021). The study noted that following the intravenous administration of *P. gingivalis*, CD4+ T cells stimulation heightened the inflammatory response among colon and lamina propria lymphocytes, resulting in an elevated Th17/Treg ratio. These results suggest that the increase in Treg cells could potentially counterbalance the escalation of intestinal tissue inflammation induced by *P. gingivalis*. However, further research is imperative to gain a comprehensive understanding of this phenomenon (Li et al., 2022). There is growing evidence suggesting a potential link between chronic inflammation, such as colitis, and an increased risk of developing colon cancer. This connection underscores the intricate interplay between inflammation caused by dysbiosis and the development of colon cancer.

Numerous investigations have shown dysbiosis-related gut microbiota associated with *F. nucleatum* infection in the tumor tissues of CRC patients (Gao et al., 2015; Dai et al., 2018; Koliarakis et al., 2019; Huh and Roh, 2020; Yu et al., 2022). The argumentative aspect revolves around determining whether *F. nucleatum* contribution to CRC is correlational or causational. Compelling evidence substantiates both hypotheses. Numerous studies on the gut microbiota have consistently demonstrated a significant prevalence of *F. nucleatum* within the tumor tissue and fecal samples of individuals with CRC (Wang et al., 2021). Etiological investigations have further elucidated the role of *F. nucleatum* as a bacterium that promotes carcinogenesis at various stages of CRC development. Two different groups were the first to show an increased abundance of this oral pathogen in CRC tissues when compared to normal tissues (Castellarin et al., 2012; Kostic et al., 2013). A recent study highlighted that identical strains of *F. nucleatum* were identified in both the saliva and colorectal tumors of patients with CRC (Komiya et al., 2019), in addition to the findings that oral *F. nucleatum* translocate to the colon through the hematogenous route (Abed et al., 2020). This suggests that CRC-associated *F. nucleatum* likely originates from the oral cavity, underscoring a potential link between oral bacteria and the development of CRC.

*Fusobacterium* is numerous in human adenomas, proposing an early role in colon carcinogenesis (McCoy et al., 2013). Kostic et al. (Kostic et al., 2013) investigated the association of *F. nucleatum* in stool and colon samples from human colorectal adenomas and adenocarcinomas. It assessed the impact of *F. nucleatum* on cancer progression and tumor-related inflammation in Apc^Min/+^ mouse model of intestinal tumorigenesis. The bacterium amplified tumor numbers and led to accelerated tumorigenesis in both the small intestine and colon, infiltration of distinct myeloid cell subsets into the tumors, and an NF-κB-driven proinflammatory profile, like F. nucleatum-positive colorectal carcinomas in humans. APC gene mutations typically manifest as early molecular alterations during the transition of epithelial cells into adenomas (Kostic et al., 2013). Hence, probably the early somatic mutations are responsible for tumor initiation, which occur before *F. nucleatum* accumulation in the tissue. This concept is further substantiated by the mechanisms described by which this bacterium contributes to CRC in the study by Rubinstein et al, where they showed that *F. nucleatum* through virulence factor FadA adhesin can adhere and invade tumor cells, and promote oncogenic and inflammatory responses to promote progression of CRC (Rubinstein et al., 2013).

Another study using the same Apc^Min/+^ mouse model showed that *F. nucleatum* induced DNA damage and cell growth in CRC by activating the E-cadherin/β-catenin pathway (Guo et al., 2020). These genetic alterations play a role in disruption of the epithelial barrier and the mucous layer facilitating the infiltration of *F. nucleatum* and other opportunistic bacteria, allowing them to establish themselves within the tumor microenvironment. However, it is essential to acknowledge that the murine model utilized lacks full characterization, potentially limiting its ability to entirely recapitulate all aspects of *F. nucleatum*-associated CRC observed in humans. These collective findings suggest that *F. nucleatum* infection has been implicated as an added environmental risk factor for CRC by promoting a proinflammatory microenvironment conducive to the progression of colorectal neoplasia. Moreover, the high abundance of *F. nucleatum* in CRC is associated with poorer survival(Flanagan et al., 2014; Mima et al., 2016; Komiya et al., 2019) and recurrence after chemotherapy (Yu et al., 2017).

There are three major virulence factors that contribute to the promotion of CRC: LPS, the adhesin FadA, and autotransporter protein Fap2 (Kostic et al., 2013; Abed et al., 2016; Lee et al., 2019). Fap2 operates as an inhibitor, dampening the tumor-killing effectiveness of T cells and NK cells. It achieves this by directly triggering the inhibitory receptor known as T-cell immunoglobulin and immunoreceptor tyrosine-based inhibitory motif domain (TIGIT), thereby contributing to a mechanism of tumor-immune evasion (Gur et al., 2015). Furthermore, Fap2 exhibits binding affinity towards a carbohydrate structure, specifically D-galactose-β(1–3)-N-acetyl-D-galactosamine (Gal-Gal/NAc), which is prominently present in CRC. Moreover, metabolic specialization may underlie the distinctive impact of *F. nucleatum* within the tumor milieu. As an asaccharolytic bacterium, it refrains from competing for glucose, a favored substrate in cancer metabolism, thereby conferring upon it a competitive advantage distinct from other microbes residing in the tumor microenvironment (Flynn et al., 2016).

Additionally, Fap2 triggers the release of proinflammatory cytokines, namely IL-8 and C-X-C motif chemokine ligand 1 (CXCL1), which enhances the migration of cancer cells (Casasanta et al., 2020). This effect contributes to the promotion of CRC cell invasiveness and potentially impacts disease progression. Synergically, LPS induces the secretion of a range of inflammatory cytokines, including IL-2, IL-6, IL-8, IL-10, IL-17, TNF- α, and upregulates NF-κB levels. On the other hand, FadA facilitates the attachment to epithelial and endothelial cells while also triggering inflammation. This interaction facilitates the attachment of *F. nucleatum* to CRC cells through the hematogenous route, contributing to the association between the oral pathogens and colon carcinogenesis (Abed et al., 2016). Furthermore, this adhesin activates pro-carcinogenic pathways directly within colon cancer cells, specifically by initiating E-cadherin-β-catenin signaling (Ito et al., 2015; Nosho et al., 2016; Rubinstein et al., 2019). In summary, *F. nucleatum* plays a role in promoting the initiation and progression of CRC through various mechanisms, including its localization within the colorectal environment, its ability to proliferate, promote immune suppression, facilitation of metastasis, and contribution to chemoresistance. These multifaceted effects collectively contribute to the complex relationship between *F. nucleatum* and CRC development.

*F. nucleatum* and *P. gingivalis* are involved in the direct activation of inflammation and carcinogenesis through multiple pathways. Although the precise role of these oral pathogens in the carcinogenesis of colon cancer is still unclear (Castellarin et al., 2012). Some studies suggest that *P. gingivalis* and *F. nucleatum* may directly interact and attach with intestinal cells and can disrupt the integrity of the epithelial barrier lining the colon. This disruption can lead to increased permeability of the barrier, allowing other opportunistic bacteria and their byproducts to enter the underlying tissues and alter the microbial communities in the gut (**Figure 2F**). This promotes chronic inflammation creating a favorable environment for cancer development and progression. These bacteria also stimulate angiogenesis, the formation of new blood vessels, which is essential for tumor growth as it supplies nutrients and oxygen to cancer cells, contributing to the growth and spread of cancer cells, bacterial metabolites, and inflammatory factors. Furthermore, they interact directly with colon cells, promoting signaling pathways that encourage cell survival, proliferation, and resistance to cell death – all hallmarks of cancer cells (Fulbright et al., 2017).

In response to the abnormal growth of colonic cells and the oral pathogens infection, a diverse array of immune cells are recruited to the tumor site (**Figure 2G**). Among them are tumor-infiltrating lymphocytes (TILs), including CD4+ helper T cells and CD8+ cytotoxic T cells, pivotal for recognizing and combating cancer cells. Macrophages, exhibiting both pro-inflammatory (M1) and anti-inflammatory (M2) functions, influence tumor progression and immune responses. DCs play a crucial role by capturing antigens and initiating immune reactions. NK cells directly target and eliminate cancer cells, restricting tumor growth. Conversely, myeloid-derived suppressor cells (MDSCs) can hinder immune responses by inhibiting T cell activity, allowing tumors to evade immunity. The intricate interplay and functions of these immune cells within the tumor context remain a focal point of research, offering potential avenues for innovative cancer treatments (Yin et al., 2020).

*P. gingivalis* is often recognized as highly proficient in evading and undermining the immune system, employing various tactics to elude and undermine immune defenses. While possessing an array of virulence factors, including gingipains, LPS, and fimbriae, *P. gingivalis* is known for invading epithelial, fibroblast, endothelial and specific immune cells. Leukocytes in circulation may function as a “Trojan horse” facilitating the oral bacterial dissemination through the bloodstream and spreading to the gut (Carrion et al., 2012). A recent review described the potential mechanisms of translocation of *P. gingivalis* from the oral mucosa by circulatory dissemination, like the Trojan Horse mechanism (de Jongh et al., 2023). A study showed that a *P. gingivalis* strain was able to significantly avoid phagocytosis and escape from macrophages (Werheim et al., 2020). Aside from macrophages, DCs are the other phagocytic cells that may play a role in blood dissemination of *P. gingivalis* (de Jongh et al., 2023). One mechanism is by engaging its fimbrial proteins with complement receptor 3 (CR3). CR3 is a major receptor for the phagocytosis of opsonized particles (Carrion et al., 2012). The clinical significance of this study (Carrion et al., 2012) lies in the remark that *P. gingivalis* strains expressing Mfa1+ infect DCs within oral mucosal tissues and the bloodstream of individuals with periodontitis. Subsequently, these infected DCs can spread to distant sites where angiogenesis occurs. El-Awady et al. investigated the mechanism of autophagy evasion by *P. gingivalis* promoting the survival within human monocyte-derived dendritic cells (MoDCs). This process is facilitated by its glycoprotein fimbriae, specifically Mfa-1, which interacts with the C-type lectin DC-SIGN found on DCs. The other primary fimbriae, known as FimA, is a TLR2 agonist targets thereby inhibit the autophagic degradation of *P. gingivalis*, which may also facilitate its dissemination to distant sites within DCs (El-Awady et al., 2015). Furthermore, another investigation revealed that macrophages subjected to differentiation in the presence of IL-34, a predominant cytokine in the oral gingival environment, exhibit significantly diminished capacity to eliminate engulfed *P. gingivalis* (Almarghlani et al., 2022). In conclusion, *P. gingivalis* exhibits a remarkable capacity to evade immune defenses and establish persistence within diverse cell types, notably DCs and macrophages. Nevertheless, the specific role of *P. gingivalis* on intestinal inflammation has yet to be fully investigated. Additionally, the precise mechanisms of translocation from the oral mucosa to the gut remain unclear.

**Figure 3:**
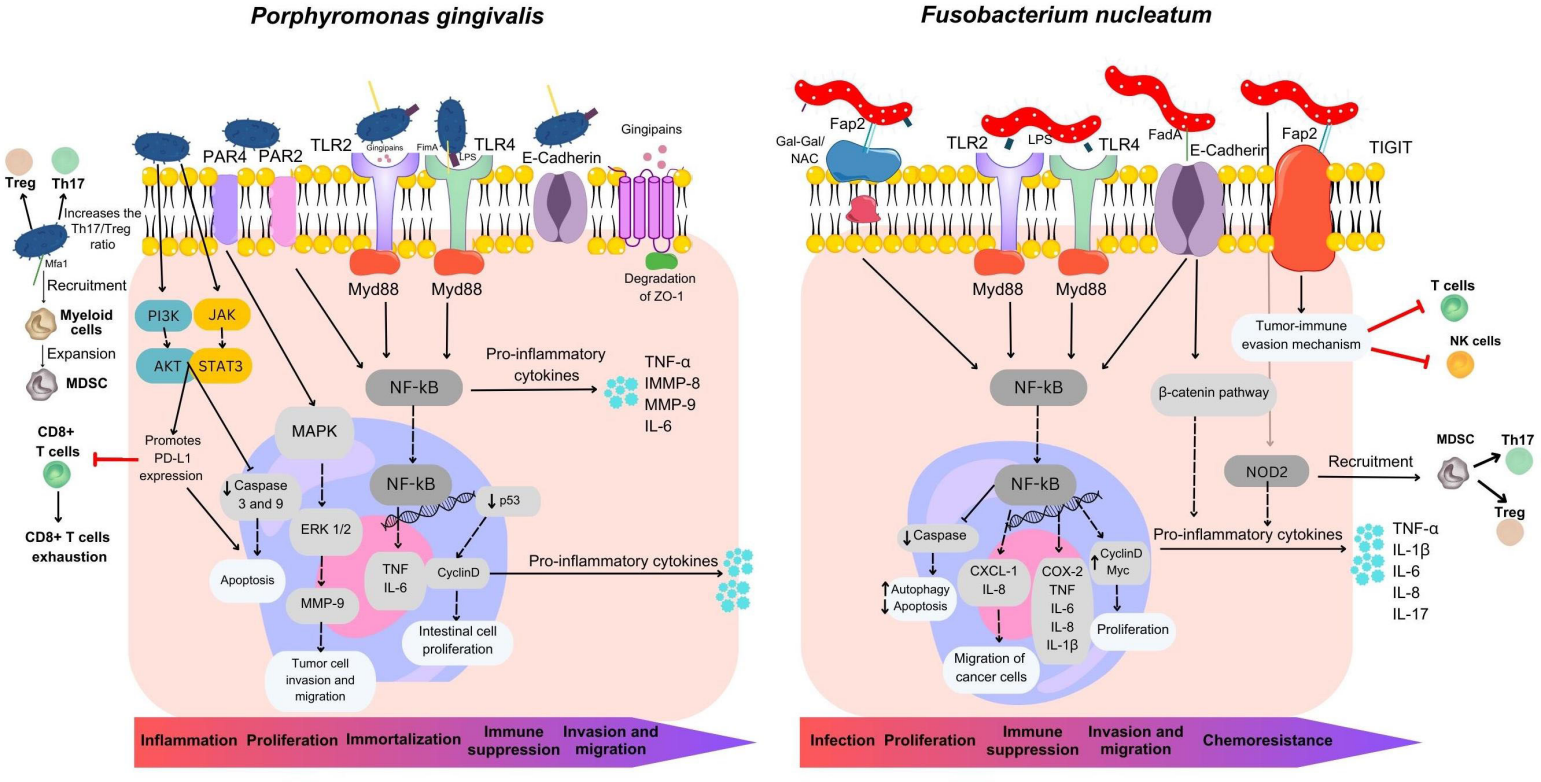
Overview of mechanisms utilized by *Fusobacterium nucleatum* and *Porphyromonas gingivalis* in colorectal carcinogenesis. *F. nucleatum* plays a multifaceted role in CRC development, contributing to its localization within the colorectal environment, proliferation, immune suppression, metastasis, and chemoresistance. It possesses three major virulence factors: Fap2, FadA, and LPS. Fap2 acts as an inhibitor, suppressing the anti-tumor activity of T and NK cells by engaging the inhibitory receptor TIGIT, facilitating tumor immune evasion. Fap2 also binds to the CRC-associated carbohydrate structure Gal-Gal/NAc, promoting attachment, and induces proinflammatory cytokine release, enhancing cancer cell migration. FadA invades and activates pro-carcinogenic pathways in colon cancer cells. *F. nucleatum*’s LPS stimulates inflammatory cytokine production, establishing a pro-inflammatory microenvironment driving CRC progression. It also targets caspase activation via NOD2, activating the IL-17F/NF-κB pathway, leading to intestinal damage, increased cytokine expression, and MDSC recruitment. *P. gingivalis* employs multiple processes to drive inflammation, cancer cell growth, invasion, metastasis, and immune suppression. It activates NF-κB and MMP-9 through gingipain proteases, promoting cancer cell invasion. Mfa1 fimbria induces oncogenic signaling and immune-suppressive MDSC expansion. Interactions with other oral bacteria lead to inflammatory cytokine release via the TLR4/NF-κB pathway. *P. gingivalis* enhances cell survival via PI3K/AKT and JAK/STAT3 while inhibiting caspase activity, modulating cell proliferation through various pathways, and manipulating immune responses by promoting PD-L1 expression and suppressing CD8+ T-cell activity via Akt-STAT3 signaling. These mechanisms collectively contribute to cancer progression.

Despite a continuous influx of numerous oral bacteria into the gastrointestinal tract, the impact of periodontal pathogens on intestinal inflammation remains as an uncharted territory (Tsuzuno et al., 2021). In **Figure 3** we depicted a comprehensive landscape of *P. gingivalis* and *F. nucleatum* virulence factors in the development and progression of CRC. Gingipain proteases produced by *P. gingivalis* can activate NF-κB and MMP-9, both significant for tumor invasion and metastasis. Furthermore, proteins associated fimbriae aid in the formation of biofilms, as well as the invasion and dissemination of the bacteria through blood DCs. Notably, Mfa1 fimbriae have been demonstrated to promote oncogenic signaling, recruiting tumor-infiltrating myeloid cells and promoting the expansion of immune-suppressive MDSC. Additionally, FimA can interact with *Streptococci, Actinomyces*, and *Treponema* spp and promote the release of the inflammatory cytokines TNF−α, IL−6, metalloproteinase-8 (MMP−8) and MMP−9 via the TLR4/NF−κB signaling pathway (Hoare et al., 2019). *P*. gingivalis binds to protease activated receptor and cleaves the MMP-9 active form, which subsequently facilitates tumor cell invasion and migration (Hoare et al., 2019; Yu et al., 2022). *P. gingivalis* displays antiapoptotic properties within epithelial cells through various mechanisms. These include the enhancement of both the PI3K/AKT and JAK/STAT3 signaling pathways, alongside the inhibition of caspase 3 and caspase 9 activity which are the final stage of the pathways of apoptosis. Additionally, *P. gingivalis*-triggered Akt–STAT3 signaling facilitates the expression of programmed cell death ligand 1 (PD-L1) while dampening CD8+ T-cell functionality, presenting another potential immune mechanism employed by the bacterium to further cancer progression (Yu et al., 2022). Also, *P. gingivalis* increases cell proliferation by modifying the activity of p53, cyclins and the WNT/β-catenin and MAPK/ERK pathways (Mu et al., 2020). Therefore, these results offer a valuable understanding of the multiple mechanisms employed by *P. gingivalis* to suppress the immune response. By elucidating the complex components and identify specific molecular targets of *P. gingivalis*-mediated immunosuppression, we can develop innovative strategies to augment the immune response that can be leveraged to enhance the effectiveness of immunotherapeutic approaches in colon cancer, ultimately benefiting patient outcomes.

Other oral bacteria, such as *T. denticola* and *T. forsythia* can also trigger the death of epithelial cells. Damaged cells release chemical signals in the form of chemokines, cytokines, and pro-inflammatory molecules that attract immune cells to the site, creating a specific environment for both innate and adaptive immune responses including MDSCs recruitment. If the cause of this process is not resolved, it can lead to chronic inflammation and persistent tissue damage (Jun et al., 2017). Both periodontitis and colon cancer have been associated with elevated MDSC levels, and increased levels of TNF-α, IL-1β, IL-6 and MMP-9 are also common factors in both conditions. In individuals without underlying health issues, hematopoietic stem cells undergo a developmental process, progressing into immature myeloid cells (IMCs). These IMCs can subsequently differentiate into granulocytes, monocytes, or mature into macrophages or DCs. In contrast, cancer patients experience a disruption in the maturation of IMCs, resulting in an elevated population of MDSCs. Importantly, both *F. nucleatum* and *P. gingivalis* have been demonstrated to induce the recruitment and proliferation of MDSCs (Kostic et al., 2013; Nosho et al., 2016; Sakamoto et al., 2021). These cells can suppress the activity of T cells, leading to T cell exhaustion and impaired anti-tumor immune responses (Cui et al., 2021). A previous report by Chen at al (Chen et al., 2020) found that the expression of NOD2, a gene coding for proteins associated with MDSCs, was upregulated in intestinal epithelial cells infected with *F. nucleatum*. *F. nucleatum* directs its actions towards caspase activation via NOD2, subsequently triggering the IL-17F/NF-κB pathway both *in vivo* and *in vitro* models. This cascade of events leads to damage to the intestinal epithelium and the upregulation of IL-1β, IL-6, IL-17F, and TNF-α (Chen et al., 2020). The activation of the TLR4/NF-κB signaling pathway by gingipain proteases and FimA, bacterial proteins produced by *P. gingivalis*, leads to an increased release of inflammatory cytokines which can have implications for the expansion and activation of MDSCs (Cai et al., 2019). Activated MDSCs within the tumor microenvironment can suppress both innate and adaptive immune responses in several ways. MDSCs inhibit the activity of T cells and NK cells and increase Treg numbers. MDSCs can also release reactive oxygen and nitrogen species, which can damage T cell receptors and interfere with T cell signaling (**Figure 3**) (Tang et al., 2021).

Based on the evidence, we propose that tumors exhibit elevated expression of genes related to MDSCs when co-infected with *F. nucleatum* and *P. gingivalis*. This co-infection synergistically enhances the presence of MDSCs within the tumor microenvironment, promoting the secretion of pro-inflammatory cytokines, producing sustained chronic inflammation, and leading to intestinal damage. These effects can lead to augmented gut permeability, enabling the infiltration of additional pathogens into the underlying tissues, thereby promoting the development of tumors.

MDSCs can also express PD-L1, which binds to PD1 on T cells, and causes secretion of IL-10 and TGF-β, which stimulate Treg activation and expansion (Tang et al., 2021). T cell exhaustion is a state of dysfunction characterized by the progressive loss of effector functions and sustained expression of inhibitory receptors, such as PD-1. Exhausted T cells are less capable of recognizing and eliminating cancer cells effectively. Targeting the recruitment and activity of MDSCs holds promise as a potential therapeutic strategy to alleviate T cell exhaustion and enhance anti-tumor immune responses. Through the suppression of MDSC recruitment or function, there exists a potential opportunity to recover T cell activity and support the role of the immune system in identifying and eradicating cancerous cells. This could involve targeting specific molecules or signaling pathways involved in the recruitment process. *F. nucleatum* and *P. gingivalis* have been shown to recruit MDSCs, leading to T cell exhaustion and impaired anti-tumor immune responses. Further research is needed to identify specific molecular targets and develop effective therapeutic interventions aimed at disrupting the immunosuppressive effects of MDSCs in the context of CRC and other cancers influenced by these bacteria.

Studying the link between oral bacteria and CRC also provides opportunities for prevention strategies. Promoting good oral hygiene practices and maintaining oral health may reduce the colonization and translocation of oral bacteria to the gut. Additionally, strategies that target the gut microbiota composition, such as dietary modifications or probiotic supplementation, can help create an environment that is less favorable for the growth and activity of these bacteria. As periodontitis is polymicrobial, mixed species potentially promote carcinogenesis both locally and in extraneous tissues, likely through complex microbial interactions. While mono-species effects on tumorigenesis pathways are elucidated, further research is required to understand the combined impact of oral species on carcinogenesis. Nevertheless, further research is needed to establish a clearer temporal relationship between *F. nucleatum* and CRC, which may be elucidated through prospective studies. By investigating the specific mechanisms by which *F. nucleatum* and *P. gingivalis* promote carcinogenesis, the field can not only develop sensitive and specific diagnostic tests that can aid in early detection and timely intervention but also identify potential therapies that specifically inhibit the activity of these bacteria or disrupt their interactions with host cells.

## Conclusions and future perspectives

In conclusion, the oral-gut-circulation axis emerges as a dynamic model for host-microbial interactions, extending its influence well beyond the oral and gut systems including the circulatory system as an essential component. The oral cavity and gastrointestinal tract house the largest and most diverse microbial communities in the human body. Recent research has revealed that these systems are not isolated but rather interact dynamically, shaping each other’s composition and function. The circulatory system, as a conduit for pathogens, bacterial products, immune cell trafficking, and inflammatory factors, adds a layer of complexity to the oral-gut axis. Strategies aimed at mitigating systemic inflammation and circulatory factors may hold therapeutic promise in this context.

Moreover, there is an emerging link between oral microbiota and cancer susceptibility. Individuals diagnosed with periodontitis exhibit a significantly elevated colon cancer risk. Thus, maintaining oral health and treating oral infections such as periodontitis may hold the key to reducing the incidence of several conditions, including colon cancer. Based on the literature that shows the tumor-promoting potential of *F. nucleatum* in CRC, treatment targeting the reduction of *Fusobacterium* populations, particularly in the oral cavity where they are notably abundant and play an essential role in biofilm formation and structure, could potentially serve as a strategy to delay or prevent tumor progression in individuals at elevated CRC risk. Additionally, *P. gingivalis*, has emerged as another player in colon cancer pathogenesis. Together their ability to stimulate a pro-inflammatory milieu within the tumor microenvironment, modulate gut microbiota, and promote angiogenesis highlights their significance. Targeting these pathogens or their virulence factors could provide novel therapeutic avenues.

The development of non-invasive screening and diagnostic tools based on microbial signatures and inflammatory markers could significantly enhance current CRC screening practices. Traditional methods, such as colonoscopy or fecal occult blood tests, although effective, may face challenges related to patient compliance, invasiveness, and cost. Salivary and blood biomarkers, on the other hand, could provide a convenient, patient-friendly, and cost-effective alternative. Additional research is needed in prevention approaches such as personalized medicine based on microbiota profiles, probiotics for gut health restoration, and lifestyle modifications as non-invasive tools for the benefit of public health to decrease the incidence of conditions such as cancer.

Fostering multidisciplinary collaboration among experts from diverse fields will be instrumental in advancing our understanding of colon cancer and the development of immunotherapeutic interventions that target, for example, specific oral pathogens. In summary, the oral-gut-circulatory axis represents a promising frontier in colon cancer research, potentially reshaping our conception of this disease and opening innovative opportunities for prevention and treatment.

## Author contributions

ST: Writing – original draft, Writing – review & editing, Conceptualization, Visualization. MA: Writing – review & editing. LM: Writing – review & editing, Supervision.
